# The relationship between obesity and disease relapse in systemic lupus erythematosus: a retrospective cohort study using the LUNA registry

**DOI:** 10.3389/fimmu.2026.1781933

**Published:** 2026-04-16

**Authors:** Naoki Suzuki, Ryusuke Yoshimi, Hiroki Mizuno, Chiharu Hidekawa, Natsuki Sakurai, Yuji Yoshioka, Daiga Kishimoto, Kaoru Takase-Minegishi, Shigeru Ohno, Nobuyuki Yajima, Ken-ei Sada, Yoshia Miyawaki, Kunihiro Ichinose, Toshimasa Shimizu, Ayuko Takatani, Hiroshi Kajiyama, Shuzo Sato, Yasuhiro Shimojima, Michio Fujiwara, Akira Onishi, Takashi Kida, Yusuke Matsuo, Keisuke Nishimura, Takahisa Onishi, Masao Katsushima, Kunihiko Umekita, Hirofumi Miyake, Hiroto Nakano, Tomoaki Ida, Kei Ikeda, Hideaki Nakajima

**Affiliations:** 1 Department of Stem Cell and Immune Regulation, Yokohama City University Graduate School of Medicine, Yokohama, Japan; 2 Clinical Laboratory Department, Yokohama City University Hospital, Yokohama, Japan; 3 Center for Rheumatic Diseases, Yokohama City University Medical Center, Yokohama, Japan; 4 Division of Rheumatology, Department of Medicine, Showa Medical University, Tokyo, Japan; 5 Department of Clinical Epidemiology, Kochi Medical School, Kochi University, Nankoku, Japan; 6 Department of Nephrology, Rheumatology, Endocrinology and Metabolism, Okayama University Graduate School of Medicine, Dentistry and Pharmaceutical Sciences, Okayama, Japan; 7 Department of Rheumatology, Shimane University Faculty of Medicine, Izumo, Japan; 8 Department of Immunology and Rheumatology, Division of Advanced Preventive Medical Sciences, Nagasaki University Graduate School of Biomedical Sciences, Nagasaki, Japan; 9 Rheumatic Disease Center, Sasebo Chuo Hospital, Sasebo, Japan; 10 Department of Rheumatology and Applied Immunology, Faculty of Medicine, Saitama Medical University, Saitama, Japan; 11 Department of Rheumatology, Fukushima Medical University School of Medicine, Fukushima, Japan; 12 Department of Medicine (Neurology and Rheumatology), Shinshu University School of Medicine, Matsumoto, Japan; 13 Department of Rheumatology, Yokohama Rosai Hospital, Yokohama, Japan; 14 Department of Advanced Medicine for Rheumatic Diseases, Graduate School of Medicine, Kyoto University, Kyoto, Japan; 15 Inflammation and Immunology, Graduate School of Medical Science, Kyoto Prefectural University of Medicine, Kyoto, Japan; 16 Department of Rheumatology, Tokyo Kyosai Hospital, Tokyo, Japan; 17 Department of Rheumatology, Graduate School of Medical and Dental Sciences, Institute of Science Tokyo, Tokyo, Japan; 18 Department of Rheumatology and Clinical Immunology, Kobe University Graduate School of Medicine, Kobe, Japan; 19 Department of Rheumatology, Kakogawa Central City Hospital, Kakogawa, Japan; 20 Department of Clinical Immunology, Osaka Metropolitan University Graduate School of Medicine, Osaka, Japan; 21 Division of Respirology, Rheumatology, Infectious Diseases, and Neurology, Department of Internal Medicine, Faculty of Medicine, University of Miyazaki, Miyazaki, Japan; 22 Department of General Internal Medicine, Tenri Hospital, Tenri, Japan; 23 Department of Rheumatology, Yokohama Minami Kyosai Hospital, Yokohama, Japan; 24 Department of Allergy and Clinical Immunology, Chiba University Hospital, Chiba, Japan; 25 Department of Rheumatology, Dokkyo Medical University, Tochigi, Japan

**Keywords:** LUNA registry, obesity, overweight, relapse, remission, systemic lupus erythematosus

## Abstract

**Objectives:**

Recent reports have suggested an association between the pathogenesis of systemic lupus erythematosus (SLE) and various adipokines secreted by adipocytes. However, the impact of obesity on the susceptibility to SLE relapses remains unclear. This study investigated the association between obesity and relapse in patients with SLE using a large, multicenter cohort.

**Methods:**

Patients enrolled in LUNA, a nationwide, multicenter SLE registry in Japan, with a body mass index (BMI) ≥ 18.5 kg/m^2^ were included in the study. Participants were divided into two groups: the obese group (BMI ≥ 25 kg/m^2^) and the normal weight group (18.5 kg/m^2^ ≤ BMI < 25 kg/m^2^). The one-year relapse rate after LUNA registration was compared between the two groups. Relapses were defined as an increase in glucocorticoid dosage during follow-up. A multivariate analysis was performed using generalized linear models adjusted for age, sex, disease duration, education level, glycated hemoglobin level, disease activity (Systemic Lupus Erythematosus Disease Activity Index), organ damage (Systemic Lupus International Collaborating Clinics/American College of Rheumatology Damage Index), current prednisolone (PSL) dose, past maximum PSL dose, calcineurin inhibitor use, and incretin-related drug use as covariates. Furthermore, a sensitivity analysis was performed using the same statistical model and covariate adjustments to reclassify participants into the overweight (BMI ≥ 25 kg/m^2^ and < 30 kg/m^2^) and non-overweight (BMI ≥ 18.5 kg/m^2^ and < 25 kg/m^2^ or BMI ≥ 30 kg/m^2^) groups.

**Results:**

Among the 1, 134 patients, 909 were classified as normal weight and 225 as obese. The multivariate analysis showed that obesity significantly reduced relapse rates (odds ratio [OR] 0.52, 95% confidence interval [CI] 0.30-0.90, *p* = 0.02). The sensitivity analysis revealed that overweight was an independent factor associated with a reduced risk of SLE relapses, with an OR lower than that for obesity in the primary analysis (OR 0.43, 95% CI 0.22–0.82, *p* = 0.01).

**Conclusion:**

Patients with mild obesity had lower relapse rates than those with normal BMI. The findings suggest that metabolic status may influence the disease course of SLE. Appropriate weight management guidance, including avoiding excessive dietary restriction, may help prevent SLE relapses.

## Introduction

Systemic lupus erythematosus (SLE) is a chronic inflammatory autoimmune disease that affects multiple organ systems (1). The management of SLE aims not only to suppress disease activity but also to maintain long-term remission, which is critical for preventing disease flares and irreversible organ damage (2). Several factors, including immunological abnormalities and metabolic status, have been implicated in the disease activity and relapse (3).

Obesity induces functional alterations in adipocytes, thereby promoting a chronic inflammatory state (4). These changes are characterized by altered adipocytes, which increase the production of pro-inflammatory adipokines and suppress the production of anti-inflammatory adipokines (4). Additionally, obesity is known to negatively impact disease activity in conditions such as rheumatoid arthritis and inflammatory bowel disease (5, 6). Studies investigating the association between SLE and adipokines have shown that leptin, an adipokine, is elevated in the serum of patients with SLE (7, 8). Furthermore, obesity is associated with an impaired ability to perform activities of daily living, increased fatigue, and increased levels of CRP and IL-6 in patients with SLE (9, 10). These findings suggest that metabolic dysregulation, including obesity and adipokine imbalance, contributes to the disease burden associated with SLE.

On the other hand, it is widely recognized that obesity reduces mortality in patients with chronic diseases such as heart failure (11–13). This fact suggests that obesity does not necessarily worsen the prognosis of SLE. However, to the best of our knowledge, all previous reports on the association between obesity and SLE were cross-sectional studies with small sample sizes (9, 10). Moreover, obesity was defined as a body mass index (BMI) of 30 kg/m^2^ or higher in these studies, but the association may vary with the degree of obesity. This study aimed to examine the association between obesity and SLE relapse to identify the potential for preventing SLE relapse through proactive lifestyle interventions.

## Materials and methods

### Study design and setting

This is a retrospective cohort study using data collected from an ongoing multicenter cohort study (the Lupus Registry of Nationwide Institutions [LUNA]), which was established in 2016, to investigate the association between clinical manifestations, socioeconomic background, and outcomes in patients with SLE reported from 24 institutions in Japan. The variables, definitions, and data collection processes within the registry have been described in previous studies using the LUNA data (14–16). Briefly, LUNA includes data on patients aged 20 years or older diagnosed with SLE according to the revised 1997 American College of Rheumatology (ACR) classification criteria (17).

This study used information from electronic medical records of patients registered between 2016 and 2022. Clinical data, including laboratory results, disease activity, and treatment administered to registered patients, were collected annually. The attending physician determined the appropriate treatment for each patient.

### Study population

This study used data from patients at the time of registration and one year after registration. Patients who met any of the following criteria were excluded: 1) currently undergoing treatment for malignancy, 2) currently pregnant, 3) no BMI record at registration (BMI is calculated as weight [kg] divided by the square of height [m]), 4) no record of relapse within one year of registration, and 5) BMI at registration < 18.5 kg/m^2^.

We excluded patients with a BMI <18.5 kg/m^2^, because an underweight status may reflect malnutrition or metabolic imbalance, which could confound the relationship between BMI and disease relapse. This exclusion criterion ensured a more homogeneous baseline metabolic profile across the study groups (18).

### Exposure

The participants were classified into two BMI categories. These categories are based on the definitions provided by the Japan Society for the Study of Obesity (JASSO) and are appropriate for the Japanese population. Specifically, normal weight is defined as 18.5–25 kg/m^2^, and obesity is defined as ≥ 25 kg/m^2^ (19).

### Outcome measures

The primary outcome of this study was the relapse rate within one year of registration. Relapse was determined by reviewing the medical records of patients who had an increased glucocorticoid dosage from the last data entry date, as confirmed by the data entry staff. Therefore, increases in glucocorticoid dosage due to factors unrelated to SLE disease activity—such as infections or perioperative management—were not classified as relapses. The presence or absence of a relapse was used as a binary variable. That is, cases that experienced at least one relapse during the one year from registration were defined as “with relapse”, and cases without any relapses were defined as “without relapse”.

### Potential confounders

Based on the findings from previous studies (20) and from the clinical perspectives of rheumatologists, the following nine variables were identified as potential confounding factors: (1) age at the time of the survey, (2) sex, (3) duration of the disease since SLE diagnosis, (4) educational level (as an indicator of medication adherence), (5) glycated hemoglobin (HbA1c) level, (6) daily dose of glucocorticoid at registration (oral prednisolone [PSL] or its equivalent in mg), (7) maximum daily dose up to registration (oral PSL or its equivalent), (8) current use of calcineurin inhibitors, (9) current use of incretin-related drugs including glucagon-like peptide-1 receptor agonists (GLP-1RAs) and dipeptidyl peptidase-4 (DPP-4) inhibitors, (10) disease activity measured using the Safety of Estrogens in Lupus Erythematosus National Assessment - Systemic Lupus Erythematosus Disease Activity Index (SELENA-SLEDAI) (21), and (11) organ damage assessed using the Systemic Lupus Collaborating Clinics (SLICC)/ACR Damage Index (SDI) (22).

### Statistical analyses

In the descriptive statistics, the mean and standard deviation (SD) were used as basic statistics for continuous data, and the *t*-test was used to test the differences between the normal-weight and obese groups. For categorical data, the percentages and frequencies were used as basic statistical measures, and chi-square tests were used to test independence.

The primary analysis was based on a multiple-imputed dataset. To address uncertainty due to missing data, missing covariate values were assumed to be missing at random. Multiple imputations were performed using the nine predefined potential confounders, the exposure variable (BMI category based on JASSO’s definition), and the outcome variable (relapse), generating 50 complete datasets. Logistic regression analysis was then applied to estimate the odds ratio (OR) for one-year relapse. All the covariates were simultaneously incorporated into a model using the forced-entry method.

As a sensitivity analysis, patients were reclassified into overweight (BMI ≥ 25 kg/m2) and non-overweight groups (BMI ≥ 18.5 kg/m^2^ and < 25 kg/m^2^ or BMI ≥ 30 kg/m^2^) to evaluate the association between being overweight as defined by the World Health Organization (WHO) and relapse. Additionally, to explore the relationship between obesity severity and relapses as a *post hoc* analysis, patients were stratified into overweight (BMI ≥ 25 kg/m^2^ and < 30 kg/m^2^) and severely obese (BMI ≥ 30 kg/m^2^) subgroups, and relapse rates were compared using univariate analysis with the chi-square test. Multiple imputations were performed using the same covariates as in the primary analysis, yielding 50 complete datasets. Logistic regression analysis was conducted to estimate the OR for one-year relapse, with all the covariates entered simultaneously using the forced-entry method.

All statistical analyses were performed using SPSS V.29.0 (IBM Corp., Armonk, New York, USA). Two-sided p-values less than 0.05 were considered statistically significant.

### Ethics approval and consent to participate

This study was conducted in accordance with the principles of the Declaration of Helsinki and Good Clinical Practice guidelines and was approved by the Institutional Review Boards of Yokohama City University (B180400002 and B181100009) and each participating hospital. Written informed consent was obtained from all the patients at the time of registration in the LUNA registry. Furthermore, an opt-out system was in place for research using registry data; research information was made public, and participants were given the opportunity to opt out.

## Results

### Patient characteristics

**Figure 1:**
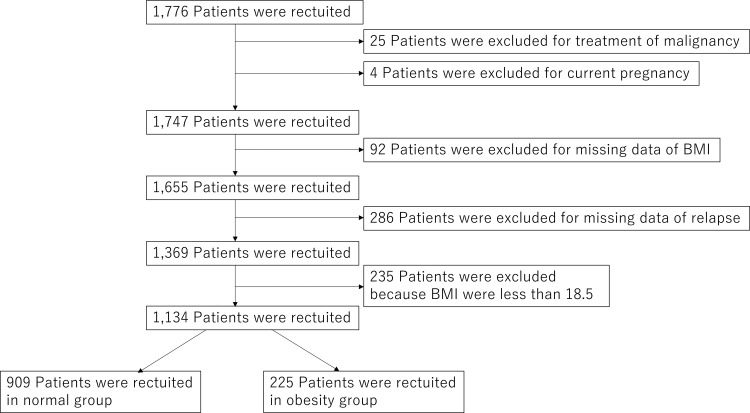
The flow chart of participant selection. Of the 1, 776 patients registered in the LUNA registry between 2016 and 2022, 25 patients undergoing treatment for malignancy and four pregnant patients were excluded at registration. Of the remaining 1, 747 patients, 378 were excluded due to missing BMI data at registration and relapse records at the 1-year follow-up, and an additional 235 were excluded due to a BMI of less than 18.5 kg/m^2^ at registration. As a result, 909 patients with a BMI of 18.5 kg/m^2^ to 25 kg/m^2^ were classified into the normal weight group, and 225 with a BMI exceeding 25 kg/m^2^ were classified into the obese group. Alt text: Flow diagram illustrating the screening process for patients in the LUNA registry between 2016 and 2022. The diagram shows sequential exclusions based on malignancy, pregnancy, missing data, and underweight status, resulting in classification into normal-weight and obese BMI groups.

Out of the 1, 776 patients registered in the LUNA registry between 2016 and 2022, 1, 134 were included in this study (**Figure 1**). Among these study participants, 909 patients (80.2%) were in the normal-weight group, and 225 (19.8%) were in the obese group. Demographic variables and clinical characteristics, including treatment status, disease activity, and immunological data, for SLE in each patient cohort are shown in **Table 1**.

**Table 1:** Baseline patient characteristics.

Variable	Normal-weight group (*n* = 909)	Obesity group (*n* = 225)	*P* value
Sex, female, *n* (%)	784 (86.2)	195 (86.7)	0.87
Age (years) ^a^	47 (15)	48 (13)	0.46
Disease duration (years) ^a^	13 (10)	13 (10)	0.64
Height (cm) ^a^	158.5 (7.3)	157.4 (7.3)	0.03
Body weight (kg) ^a^	53.6 (6.7)	71.3 (10.1)	<0.01
Final education, *n* (%)			0.02
Junior high school	38 (4.5)	19 (9.0)	
High school	604 (71.4)	149 (70.6)	
College	204 (24.1)	43 (20.4)	
SELENA-SLEDAI ^a^	5.2 (4.5)	5.4 (4.6)	0.60
SDI ^a^	1.2 (1.6)	1.5 (1.9)	0.11
Drugs			
Current GC dose (mg/day of PSL equivalent) ^a^	6.5 (5.9)	7.5 (8.3)	0.07
Past maximum GC dose (mg/day of PSL equivalent) ^a^	39.7 (38.2)	42.5 (19.9)	0.14
MMF, *n* (%)	119 (13.1)	40 (17.9)	0.06
MZB, *n* (%)	62 (6.9)	14 (6.3)	0.74
MTX, *n* (%)	32 (3.5)	2 (0.9)	0.03
AZA, *n* (%)	90 (9.9)	23 (10.3)	0.88
TAC, *n* (%)	322 (35.5)	76 (33.9)	0.65
CyA, *n* (%)	45 (5.0)	16 (7.1)	0.19
BEL, *n* (%)	25 (3.7)	7 (4.0)	0.84
HCQ, *n* (%)	290 (32)	64 (28.7)	0.34
Incretin-related drugs ^b^, *n* (%)	29 (3.2)	25 (11.2)	<0.01
Laboratory data			
White blood cells (/µL) ^a^	5,885 (2,207)	6,602 (2,516)	<0.01
Creatinine (mg/dL) ^a^	0.82 (0.81)	0.81 (0.71)	0.85
CRP (mg/dL) ^a^	0.22 (0.65)	0.41 (0.88)	<0.01
CH50 (U/dL) ^a^	37.8 (12.5)	41.3 (13.0)	<0.01
IgG (mg/dL) ^a^	1,421 (487)	1,388 (468)	0.41
HbA1c (%) ^a^	5.6 (0.5)	5.9 (0.7)	<0.01
Anti-dsDNA antibodies (IU/mL) ^a^	21 (43)	20 (47)	0.85

^a^ Values are expressed as mean (standard deviation).

^b^ Glucagon-like peptide-1 receptor agonists or dipeptidyl peptidase-4 inhibitors.

SELENA-SLEDAI, Safety of Estrogens in Lupus Erythematosus National Assessment - Systemic Lupus Erythematosus Disease Activity Index; SDI, Systemic Lupus International Collaborating Clinics/American College of Rheumatology Damage Index, GC, glucocorticoid; MMF, mycophenolate mofetil; MZB, mizoribine; MTX, methotrexate; AZA, azathioprine; TAC, tacrolimus; CyA, cyclosporine; BEL, belimumab; HCQ, hydroxychloroquine.

The proportions of women in the normal-weight and obese groups were similar (86.2% vs. 86.7%, *p* = 0.87). There were no significant differences in the mean age (47 years [SD 15] vs. 48 years [SD 13], *p* = 0.46) or mean disease duration (13 years [SD 10] vs. 13 years [SD 10], *p* = 0.64) between the two groups. However, the level of final education was significantly lower in the obese group than in the normal weight group (*p* = 0.02).

The mean PSL-equivalent dose of glucocorticoid at registration was 6.5 mg/day (SD 5.9) in the normal weight group and 7.6 mg/day (SD 8.2) in the obese group, with no significant difference. However, the mean past maximum PSL-equivalent dose of glucocorticoid was significantly higher in the obese group than in the normal-weight group (42.5 mg/day [SD 19.9] vs. 38.4 mg/day [SD 18.2], *p* < 0.01). There were no significant differences in immunosuppressant use rates between the two groups. The prevalence of incretin-related drugs (GLP-1RAs or DPP-4 inhibitors) was higher in the obese group than in the normal-weight group (11.2% vs. 3.2%, *p* < 0.01).

Regarding laboratory findings, the obese group had significantly higher white blood cell count and CRP, CH50, and HbA1c levels than the normal-weight group. There were no significant differences in serum creatinine, IgG, or anti-dsDNA antibody levels between the two groups.

The mean SELENA-SLEDAI score was 5.2 (SD 4.5) in the normal-weight group and 5.4 (SD 4.6) in the obese group, with no significant difference between the groups. Additionally, the mean SDI score was 1.2 (SD 1.6) in the normal-weight group and 1.5 (SD 1.9) in the obese group, with no significant difference between the groups. On the other hand, the relapse rate at one year was 13.3% in the normal weight group and 7.9% in the obese group; the obese group showed a significantly lower rate compared to the normal-weight group (*p* = 0.02).

### Primary analysis - association between SLE relapse and obesity

Relapse within 1 year of registration occurred in 123 individuals (13.5%) in the normal-weight group and 18 individuals (8.0%) in the obese group, with the obese group showing a significantly lower rate than the normal-weight group (*p* = 0.02). To investigate differences in relapse rates between the normal-weight and obese groups, while adjusting for patient background, we performed logistic regression analyses after imputing missing values via multiple imputation. The results identified obesity, as defined by JASSO, as an independent factor associated with a lower relapse rate (OR 0.52 [95% confidence interval (CI) 0.30-0.90], *p* = 0.02) (**Table 2**).

**Table 2:** Logistic regression model for relapse risk with obesity as the exposure variable.

Variable	OR (95% CI)	*P* value
BMI ≥ 25	0.52 (0.30-0.90)	0.02
Age	0.97 (0.95-0.98)	<0.01
Sex (female)	0.73 (0.43-1.23)	0.23
Disease duration	1.01 (0.99-1.04)	0.29
Final education	0.85 (0.59-1.24)	0.41
HbA1c	1.23 (0.81-1.87)	0.33
Current GC dose	1.02 (1.00-1.05)	0.07
Past maximum GC dose	0.99 (0.98-1.00)	0.20
Calcineurin inhibitor ^a^	1.66 (1.15-2.40)	<0.01
Incretin-related drugs ^b^	1.35 (0.50-3.68)	0.55
SELENA-SLEDAI	1.03 (0.98-1.07)	0.29
SDI	0.96 (0.83-1.11)	0.58

^a^At least one of the following: tacrolimus, cyclosporine.

^b^At least of the following classes of medications: glucagon-like peptide-1 receptor agonists, dipeptidyl peptidase-4 inhibitors.

OR, odds ratio; CI, confidence interval; GC, glucocorticoid; SELENA-SLEDAI, Safety of Estrogens in Lupus Erythematosus National Assessment - Systemic Lupus Erythematosus Disease Activity Index; SDI, Systemic Lupus International Collaborating Clinics/American College of Rheumatology Damage Index.

### Sensitivity analysis - association between SLE relapse and overweight

To clarify the extent to which overweight, as defined by WHO, reduces the risk of relapse, we divided the 1, 134 participants into the overweight group (BMI ≥ 25 kg/m^2^, < 30 kg/m^2^, *n* = 168) and the non-overweight group (BMI ≥ 18.5 kg/m^2^ and < 25 kg/m^2^ or BMI ≥ 30 kg/m^2^, *n* = 966) and compared the relapse rate of SLE at one-year follow-up. After imputing missing values using multiple imputation, logistic regression analysis identified overweight as an independent factor associated with a lower risk of SLE relapse, with an OR lower than that for obesity in the primary analysis (0.43 [95% CI 0.22-0.82], *p* = 0.01) (**Table 3**).

**Table 3:** Logistic regression model for relapse risk with overweight as the exposure variable.

Variable	OR (95% CI)	*P* value
BMI ≥ 25 kg/m^2^, < 30 kg/m^2^	0.43 (0.22-0.82)	0.01
Age	0.97 (0.95-0.98)	<0.01
Sex (female)	0.71 (0.42-1.19)	0.19
Disease duration	1.01 (0.99-1.04)	0.20
Final education	0.87 (0.60-1.27)	0.48
HbA1c	1.21 (0.81-1.81)	0.35
Current GC dose	1.03 (1.00-1.05)	0.06
Past maximum GC dose	0.99 (0.98-1.00)	0.14
Calcineurin inhibitor ^a^	1.68 (1.16-2.42)	<0.01
Incretin-related drugs ^b^	1.27 (0.81-1.81)	0.64
SELENA-SLEDAI	1.02 (0.98-1.07)	0.36
SDI	0.96 (0.82-1.12)	0.59

^a^At least one of the following: tacrolimus, cyclosporine.

^b^At least of the following classes of medications: glucagon-like peptide-1 receptor agonists, dipeptidyl peptidase-4 inhibitors.

OR, odds ratio; CI, confidence interval; GC, glucocorticoid; SELENA-SLEDAI, Safety of Estrogens in Lupus Erythematosus National Assessment - Systemic Lupus Erythematosus Disease Activity Index; SDI, Systemic Lupus International Collaborating Clinics/American College of Rheumatology Damage Index.

### *Post hoc* analysis - comparison of overweight and severe obesity in SLE relapse

We further stratified the 225 obese group participants into two groups based on BMI: the overweight group (BMI ≥ 25 kg/m^2^ and < 30 kg/m^2^, *n* = 168) and the severely obese group (BMI ≥ 30 kg/m^2^, *n* = 57), and compared the SLE relapse rates at one year using univariate analysis. The severely obese group showed a tendency toward higher SLE relapse compared to the overweight group, although no statistically significant difference was observed (12.3% [7/57] vs. 6.5% [11/168], *p* = 0.16).

## Discussion

In this study, obesity, defined as BMI ≥25 kg/m^2^, was associated with a lower risk of one-year relapse. In particular, the sensitivity analysis results suggested that overweight status, defined as BMI ≥25 kg/m^2^ and <30 kg/m^2^, may be protective against SLE relapse.

WHO defines a BMI range of 25 kg/m^2^ to 30 kg/m^2^ as overweight and a BMI ≥ 30 kg/m^2^ as obese (23). In contrast, JASSO defines obesity as a BMI ≥ 25 kg/m^2^, because the prevalence and severity of obesity remain relatively mild in Japan (19). As this clinical study was conducted in Japan, the study population was classified based on the definition of obesity provided by JASSO. Even so, the obese group constituted only about 20% of all participants. Furthermore, when this obese group was subdivided according to the WHO classification, the overweight group accounted for approximately one-quarter, with the remainder classified as obese. This background on the study population facilitated a focus on the impact of overweight in SLE, which had not previously been reported. In both the primary analysis and sensitivity analyses of this study, overweight individuals showed a lower tendency for relapse than the controls. On the other hand, in the *post hoc* analysis, although no statistically significant difference was observed, a trend toward higher relapse rates was noted in the WHO-based obese group compared to the overweight group. These results suggest that the relapse rate is lower in the overweight group than in both the normal weight and obese groups, indicating a U-shaped curve in the relationship between relapse rate and body weight.

In general, obesity increases the incidence of obesity-related complications and mortality from those complications (23). In contrast, in patients with chronic diseases such as heart failure or malignancy, obesity is a favorable prognostic factor, known as the obesity paradox (24, 25). In heart failure, myokines secreted by skeletal muscles and adipokines secreted by adipocytes are cardioprotective (24). In malignancy, it has been proposed that excess adipose tissue acts as a nutrient reservoir, conferring a survival advantage during stress (25). Such protective effects of obesity and adipokines may also exist in the pathogenesis of SLE. Among systemic autoimmune diseases, a study reported lower disease activity in obese patients than in the non-obese with Sjögren’s disease (26).

The mechanisms linking obesity to disease activity in SLE are likely multifactorial, involving complex interactions among immunometabolic pathways, inflammatory signaling, and adipokines. Among these, type I IFN signaling represents a key SLE-specific pathway that may help explain the association between obesity and disease activity. Leptin, an adipokine that increases with obesity, can suppress type I IFN response (27, 28). Mechanistically, leptin acts on immune cells to induce suppressor of cytokine signaling 3 (SOCS3), thereby suppressing the Janus kinase-signal transducer and activator of transcription (JAK-STAT) pathway and attenuating type I IFN response (27). Given that type I IFN is a key factor in SLE pathogenesis (29), lower IFN-α levels in the overweight group compared with the normal-weight group may contribute to reduced disease activity and a lower risk of relapse. However, the relationship between BMI and IFN signaling may be bidirectional. IFN-α, which is used therapeutically for viral hepatitis, causes weight loss or anorexia (30). Therefore, a low BMI may not simply represent a risk factor for disease flare or relapse mediated by reduced leptin and SOCS3 levels and enhanced type I IFN activity; rather, it may also be a consequence of excessive type I IFN signaling.

The mechanisms linking obesity to SLE disease activity cannot be explained solely by the leptin-type I IFN pathway and may be more complex. Obesity may influence the immune response by reprogramming immunometabolism; changes in cellular metabolic pathways are believed to directly affect immune cell function (31). In particular, impaired glucose metabolism in effector T cell subsets is associated with increased BMI, potentially leading to attenuated inflammatory responses and reduced disease activity (32). A previous report analyzing the characteristics of 34 SLE patients in relation to BMI similarly found that BMI was negatively correlated with SLEDAI-2K scores (33). The authors concluded that although serum IFN-α and IFN-γ levels were negatively correlated with BMI, causal mediation analysis indicated that BMI directly influences SLEDAI-2K scores independently of these factors. Immunophenotyping analysis identified inverse relationships between BMI and cellular metabolic pathways, including glucose metabolism in Th1 and effector memory CD8^+^ T cells. These findings suggest that obesity-related metabolic abnormalities may suppress effector T-cell responses, thereby regulating disease activity.

Furthermore, adipokines may exert context-dependent effects on immune regulation. Leptin is generally considered a pro-inflammatory factor and has been reported to be associated with increased Th17 cell numbers (34, 35). Moreover, although adiponectin has traditionally been recognized as an anti-inflammatory adipokine, accumulating evidence suggests that it paradoxically exacerbates inflammation in certain diseases characterized by systemic inflammatory states (36). For example, adiponectin has been reported to induce inflammation in chondrocytes and synovial fibroblasts in rheumatoid arthritis (37) and in colonic epithelial cells in Crohn’s disease (38). In patients with SLE, serum adiponectin levels are higher than in healthy individuals and have been reported to correlate positively with serum TNF-α levels (39). Obesity is associated with decreased serum adiponectin levels (40), suggesting that this reduction may contribute to the lower SLE recurrence rate observed in the overweight group. Based on these findings, obesity may suppress disease activity independently of type I IFN signaling.

Taken together, these findings suggest that obesity may influence SLE disease activity through multiple, partially independent pathways, including regulation of type I IFN signaling, alterations in T-cell immunometabolism, and complex immune modulation mediated by adipokines. These multifaceted effects likely render the association between BMI and SLE activity bidirectional and nonlinear rather than linear, supporting the notion that a moderate BMI range is associated with a more favorable disease course.

This study had some limitations. First, there are inherent limitations to inferring causality because this study was retrospective. To minimize the risk of reverse causality, we adopted a longitudinal study design and statistically adjusted for potential confounding factors to the extent possible. Given the nature of the research question, it would be practically difficult to conduct an intervention study. Moving forward, it would be desirable to validate the findings of this study in different populations through prospective observational studies or similar approaches. Second, we could not adjust for a history of disease relapse, which may have influenced the current BMI through changes in treatment intensity, such as cumulative glucocorticoid exposure. This limitation raises the possibility of reverse causality, whereby prior relapses and associated therapies may have contributed to obesity at the time of registration. However, the observed association between higher HbA1c and increased relapse risk (OR ≈ 1.46) is inconsistent with this possibility, as HbA1c, which is also influenced by glucocorticoid exposure, did not show a protective association. Third, this study did not fully eliminate the potential confounding effects of glucocorticoids. The data collection items did not include the cumulative glucocorticoid dose or the duration of administration at the time of registration. Instead, the maximum prior glucocorticoid dose and the glucocorticoid dose at registration were included as potential confounders in the multivariate analysis. This approach allowed us to exclude confounding factors related to glucocorticoids to the greatest extent possible. Fourth, the data entry worker determined relapses in the LUNA registry based on medical record information when there was an increase in glucocorticoid dose since the last entry date. Therefore, cases in which other immunosuppressants were added without an increase in glucocorticoids were not considered relapses. However, because glucocorticoids remained the primary treatment for SLE, most relapses would likely have been treated with increased glucocorticoid doses, suggesting the impact of this definition was minimal. Furthermore, increases in glucocorticoid dosage that were not clinically judged by the physician to represent a relapse were not counted as relapses. This approach likely minimized misclassification by excluding treatment adjustments made for reasons other than disease activity. However, future studies should validate these findings through prospective observational designs or confirmatory analyses using a more standardized and clinically robust definition of relapse. Fifth, this study was unable to conduct a detailed analysis of relapse types since the LUNA registry does not collect data on specific types of relapse (e.g., clinical, serological, or organ-specific). The EULAR Treat-to-Target recommendations explicitly state that treatment should not be intensified solely on the basis of serological activity (41), and the latest DORIS remission definition also use the clinical SLEDAI-2K score, which does not depend on the presence or absence of serological abnormalities (42). Moreover, the Japanese guideline also does not recommend intensifying treatment solely on the basis of serological abnormalities (43). Since SLE management in Japan generally follows these recommendations and guidelines, it is believed that there were few cases in this registry where glucocorticoid doses were increased solely due to serological relapse. Finally, the applicability of the BMI-based definitions of overweight and obesity used in this study to other ethnic groups and regions remains uncertain, potentially limiting the external validity of our findings. Nevertheless, this study is important in that it suggests that maintaining a “moderate” BMI—neither too low nor too high—is associated with a reduced risk of recurrence. Although BMI thresholds for overweight and obesity may vary across populations, similar associations may be observed in other ethnic and regional groups. Future studies involving diverse populations are needed to clarify the generalizability of this association and to identify the optimal BMI range associated with a reduced risk of SLE recurrence in each population.

Despite these limitations, this study has several strengths. First, unlike most previous studies on this topic, which were cross-sectional, our analysis employed a cohort design, enabling assessment of the temporal relationship between obesity and relapse. Second, the study included a large, well-characterized sample of over 1, 100 patients enrolled across multiple institutions, providing sufficient statistical power and enhancing the reliability of the findings. Finally, the use of multiple complementary analytical approaches, including multivariable adjustment, multiple imputation for missing data, and *post hoc* analysis, reinforces the robustness and internal validity of our results.

In conclusion, this study suggests that mild obesity is associated with a lower relapse rate. By guiding appropriate weight management that avoids excessive dieting, SLE relapse may be prevented more effectively.

## Data Availability

The datasets used and/or analyzed during the current study are available from the corresponding author on reasonable request.
